# Detection and Characterization of Low Temperature Peat Fires during the 2015 Fire Catastrophe in Indonesia Using a New High-Sensitivity Fire Monitoring Satellite Sensor (FireBird)

**DOI:** 10.1371/journal.pone.0159410

**Published:** 2016-08-03

**Authors:** Elizabeth C. Atwood, Sandra Englhart, Eckehard Lorenz, Winfried Halle, Werner Wiedemann, Florian Siegert

**Affiliations:** 1 GeoBio Center, Department of Biology II, Ludwig-Maximilians-Universität Munich, Planegg-Martinsried, Germany; 2 RSS Remote Sensing Solutions GmbH, Baierbrunn, Germany; 3 Institute of Optical Sensor Systems, German Aerospace Center (DLR e.V.), Berlin-Adlershof, Germany; Kerala Forest Research Institute, INDIA

## Abstract

Vast and disastrous fires occurred on Borneo during the 2015 dry season, pushing Indonesia into the top five carbon emitting countries. The region was affected by a very strong El Niño-Southern Oscillation (ENSO) climate phenomenon, on par with the last severe event in 1997/98. Fire dynamics in Central Kalimantan were investigated using an innovative sensor offering higher sensitivity to a wider range of fire intensities at a finer spatial resolution (160 m) than heretofore available. The sensor is onboard the TET-1 satellite, part of the German Aerospace Center (DLR) FireBird mission. TET-1 images (acquired every 2–3 days) from the middle infrared were used to detect fires continuously burning for almost three weeks in the protected peatlands of Sebangau National Park as well as surrounding areas with active logging and oil palm concessions. TET-1 detection capabilities were compared with MODIS active fire detection and Landsat burned area algorithms. Fire dynamics, including fire front propagation speed and area burned, were investigated. We show that TET-1 has improved detection capabilities over MODIS in monitoring low-intensity peatland fire fronts through thick smoke and haze. Analysis of fire dynamics revealed that the largest burned areas resulted from fire front lines started from multiple locations, and the highest propagation speeds were in excess of 500 m/day (all over peat > 2m deep). Fires were found to occur most often in concessions that contained drainage infrastructure but were not cleared prior to the fire season. Benefits of implementing this sensor system to improve current fire management techniques are discussed. Near real-time fire detection together with enhanced fire behavior monitoring capabilities would not only improve firefighting efforts, but also benefit analysis of fire impact on tropical peatlands, greenhouse gas emission estimations as well as mitigation measures to reduce severe fire events in the future.

## Introduction

The fires that swept across Indonesia during the latter half of 2015 were catastrophic on many levels. Costs incurred from the fires to the Indonesian government are estimated to be in excess of USD 16 billion [1], signifying 1.9% of the national gross domestic product. Greatly reduced air quality in Southeast Asia is a consequence of major forest fires [2–4], and the resulting smoke cloud, coined the 2015 Southeast Asian Haze, spread across several countries, including Brunei, Indonesia, Malaysia, Singapore, Southern Thailand, Vietnam, Cambodia and the Philippines. The islands of Sumatra and Borneo were especially heavily impacted, with poor air quality causing a state of emergency to be declared in six Indonesian provinces. On Borneo, the province of Central Kalimantan was severely affected with Pollutant Standards Index (PSI) of fine particulate matter (PM_2.5_) hitting recorded highs in excess of 1,500, far exceeding levels deemed hazardous for human health [5–7]. Borneo contains many tropical peatlands [8], and burning of peat swamp forests has been found to damage biodiversity [9], ecosystem structure [10] and local livelihood opportunities [11]. Initial emission estimates from the 2015 peat fires amount to 1.75 billion metric tons of CO_2_ equivalents [1], placing Indonesia as the world’s fifth highest carbon dioxide emitting country above other nations such as Japan and Germany [12,13].

Worldwide, tropical peatlands are estimated to cover an area ranging from 39–66 million hectares (ha), representing between 10–16% of global peatland resources [14,15]. Indonesia contains more than half of all known peatlands in the tropical zone, with an area ranging from 16–27 million ha [8,16] and translating to a peat carbon pool of 82–92 gigatons (GT) [14]. For millennia, Borneo has been primarily covered with tropical peatlands [8,16]. In recent decades, peat swamp forests in this region have been degraded through both industrial and illegal logging [6,17], industrial plantation activities [18,19] and infrastructure from failed development projects such as the Mega Rice Project [20,21]. Peatlands naturally have a high water table, lying at or just below the forest-covered surface [16]. Drainage infrastructure, such as canals, can contribute to lowering the water table [21–23], which is then compounded by drought periods coinciding with climatological events such as El Niño-Southern Oscillation (ENSO) [6,24–26]. The reduced water table allows drying of the peat layer, often for the first time in centuries [16], and thus becoming more susceptible to catching fire [21,22]. Fire is often utilized as a cheap, effective method to clear and maintain land for both agricultural and plantation development [27]. On Borneo, slash-and-burn techniques often result in fires spreading into surrounding un-slashed peat swamp forests [22]. Peatland fires are characterized by low intensity burning, which can spread into peat deposits up to 0.5 m below the surface [21,28], and can burn for long periods of time, often being very difficult to extinguish [22]. Smoldering peatland fires produce large amounts of particulate matter, CO and other gas compounds [22,29]. Peatland and forest fires in Indonesia during the 1997/98 ENSO event are estimated to have released 0.2–0.4 Pg C, accounting for at least 10% of the global total carbon emissions due to forest fires [30]. Conservation efforts have included the creation of national parks to slow the peatland degradation process, including the Sebangau National Park established in 2004 through a combined effort of the World Wildlife Foundation (WWF) and the Indonesian Ministry of Environment and Forestry (MoEF). Fires occurred both within the park and in neighboring regions from September-October 2015, although the extent of damage incurred remains to be clarified.

Many questions remain regarding better fire management practices to help avoid catastrophic fire events in Indonesia such as those in 1982/83, 1997/98 and recently in 2015. Remote sensing systems have been utilized for over three decades to support monitoring efforts [31–33]. Techniques using spectral bands in the visible and near infrared (VNIR) and the shortwave infrared (SWIR) are limited by smoke and haze coverage while fires are burning [34], and thus the field has focused on sensors in the midwave and thermal infrared (MWIR & TIR) to detect active fires. The latter class of sensors include NOAA-AVHRR [35–37], GOES-VAS [38], ERS-ATSR [39,40], TRMM-VIIRS [41], and MODIS on the EOS Terra and Aqua satellites [42]. These sensors offer a pixel resolution from 1 km down to 375 m, and most saturate at a relatively low brightness temperature of ca. 300–340 K, with the exception of a single MODIS band (channel 21, 3.9 μm low-gain) which saturates at 500 K. Low sensor saturation inhibits proper detection of very large fire events [43]. While MODIS is best able to overcome this limitation, the 1-km pixel resolution hinders detection of initial fire fronts or separation of multiple small fires.

The Technology Experiment Carrier (TET-1) is one of two experimental satellites in the German Aerospace Center (Deutsche Luft- und Raumfahrt, DLR) FireBird mission. The onboard sensor saturates at 900 K, improving its ability to successfully detect high-temperature events (HTE) ranging from smoldering low intensity fires to large-area high intensity fires [43]. This, together with the sensor’s 160-m spatial resolution, may result in improved active fire monitoring and allow measurement of fire dynamic behavior previously not possible.

In this paper, we explore whether TET-1 can provide improved fire detection capabilities than hereto existing systems, thus providing the basis for an improved early-detection fire management system. Focus is paid to peat fire dynamics (propagation speed, area burned) over different ground and vegetation types, as well as fire occurrence in and around concession areas. Finally, we provide a first estimate of the damage incurred to the Sebangau National Park and surrounding regions during the 2015 wildfires derived from detection algorithms for both active fire (MODIS, TET-1) and burned area (Landsat).

## Materials and Methods

### Study area and available metadata

The Sebangau National Park and surrounding areas sit upon peat layers reaching at least 9 m deep [16]. The park is home to many endemic and endangered species, including the clouded leopard, sun bear and Orang-Utan (critically endangered). Botanic biodiversity within the park comprises 106 different known species, which encompasses many orchid as well as medicinally useful plants. Prior to establishment of the park, the area was systematically logged through both industrial and illegal activities [6,44]. Neighboring the eastern boundary of the park is the location of the former Mega Rice Project. This project was initiated by the Indonesian Government in 1995 but subsequently ended three years later when recognized as a failure [20]. During this period, over 4,000 canals were constructed with the primary goal to establish land for agriculture but also succeeded in providing transport infrastructure for logs out of the forest. Through accelerating waterflow from the peatlands, this infrastructure contributed to lowering the water table and resulted in serious degradation of an area more than 1 million hectares in size [21,45]. Recent conservation efforts by the World Wildlife Foundation (WWF) include reforestation as well as building dams, with the goal to encourage a return to historical water table levels, thus preventing drying out of the peat layer [45] and reducing risk of fire [21,22].

**Fig 1:**
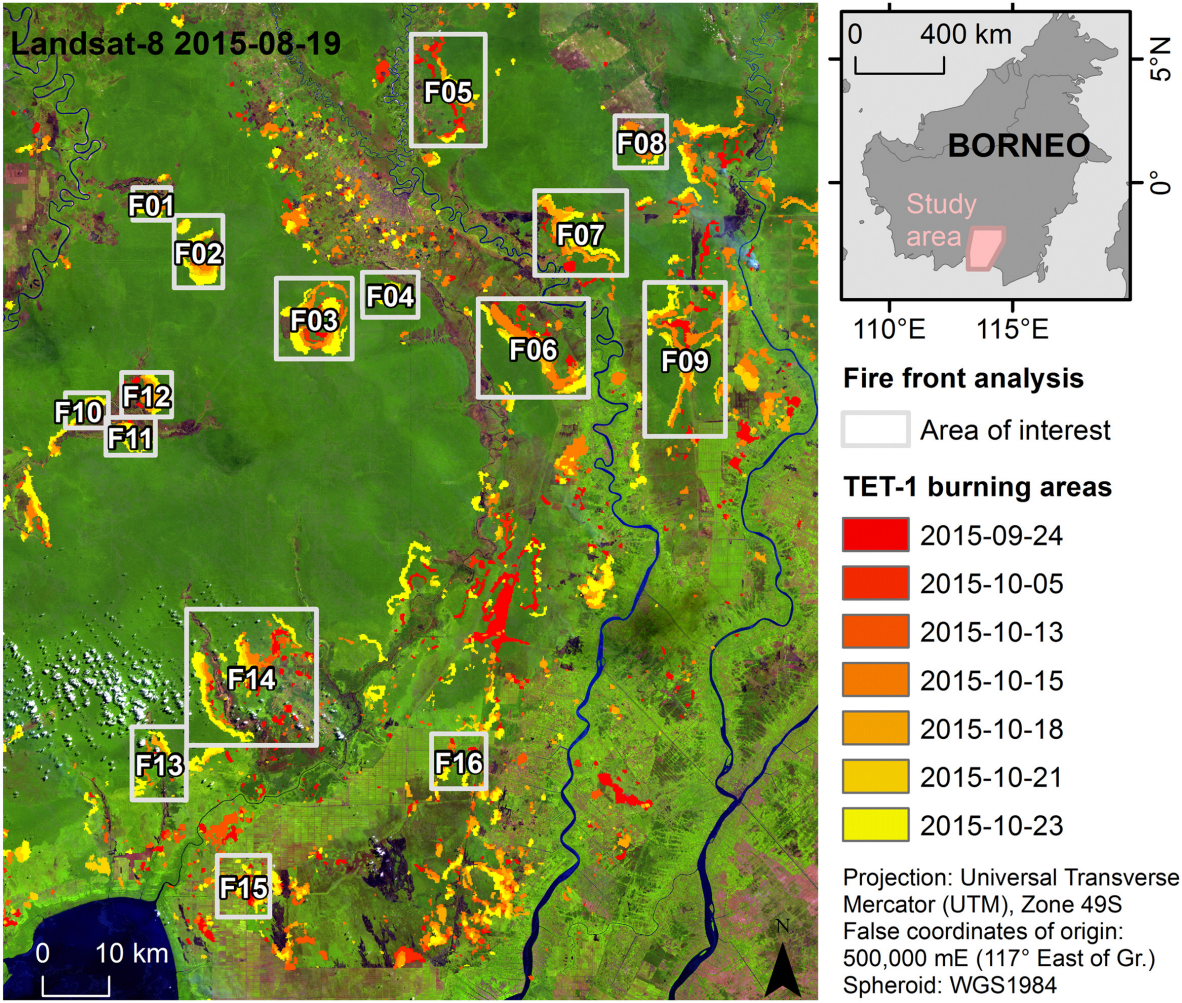
Study area and fire dynamic Areas of Interest (AOI) overview. Landsat-8 OLI image (false color: R, band 9 1.36–1.38 μm; G, band 5 0.85–0.88 μm; B, band 4 0.64–0.67 μm; source USGS/NASA) from Aug. 19^th^, 2015, overlain with TET-1 active fire classifications derived from seven acquisition dates (displayed in red to yellow). The AOI for each fire dynamic measurement area is indicated in light grey and study area location in Central Kalimantan, Borneo, is indicated in the inset.

The study area (Fig 1) was selected to cover the Sebangau National Park as well as neighboring oil palm concessions and degraded areas, and extended over 2,430,390 ha. Datasets for peat depth, primary forest cover and known plantation concessions were accessed from Global Forest Watch [46]. Peat depth data, covering both Indonesia and Malaysia, were made available by the Indonesian Ministry of Agriculture. Based upon these data, we separated the study area into regions of thin peat coverage over sand and the available peat depth classes: 0–1 m, 1–2 m and more than 2 m.

Unburned forest coverage, defined as having not burned within the last 30 years and created using a Landsat time series covering 2000–2012 [44], was used to make an initial separation of vegetation types. Additional land cover classification maps covering the time period 1990–2013 produced by MoEF were downloaded from Greenpeace [47]. The land cover data allowed further separation of previously burned areas into “swamp scrubland”, covered by bushes and brush, and “swamp”, covered primarily by grass and sedges. Unburned forest was termed “secondary swamp forest” to match the categories provided in the land cover dataset. Visual separation of unburned forest into mixed swamp, low pole and tall interior forest types was accomplished based on previous studies in the area [16], and analysis of Landsat-8 OLI imagery from August 2015 and Landsat-5 TM imagery from June 1991.

**Fig 2:**
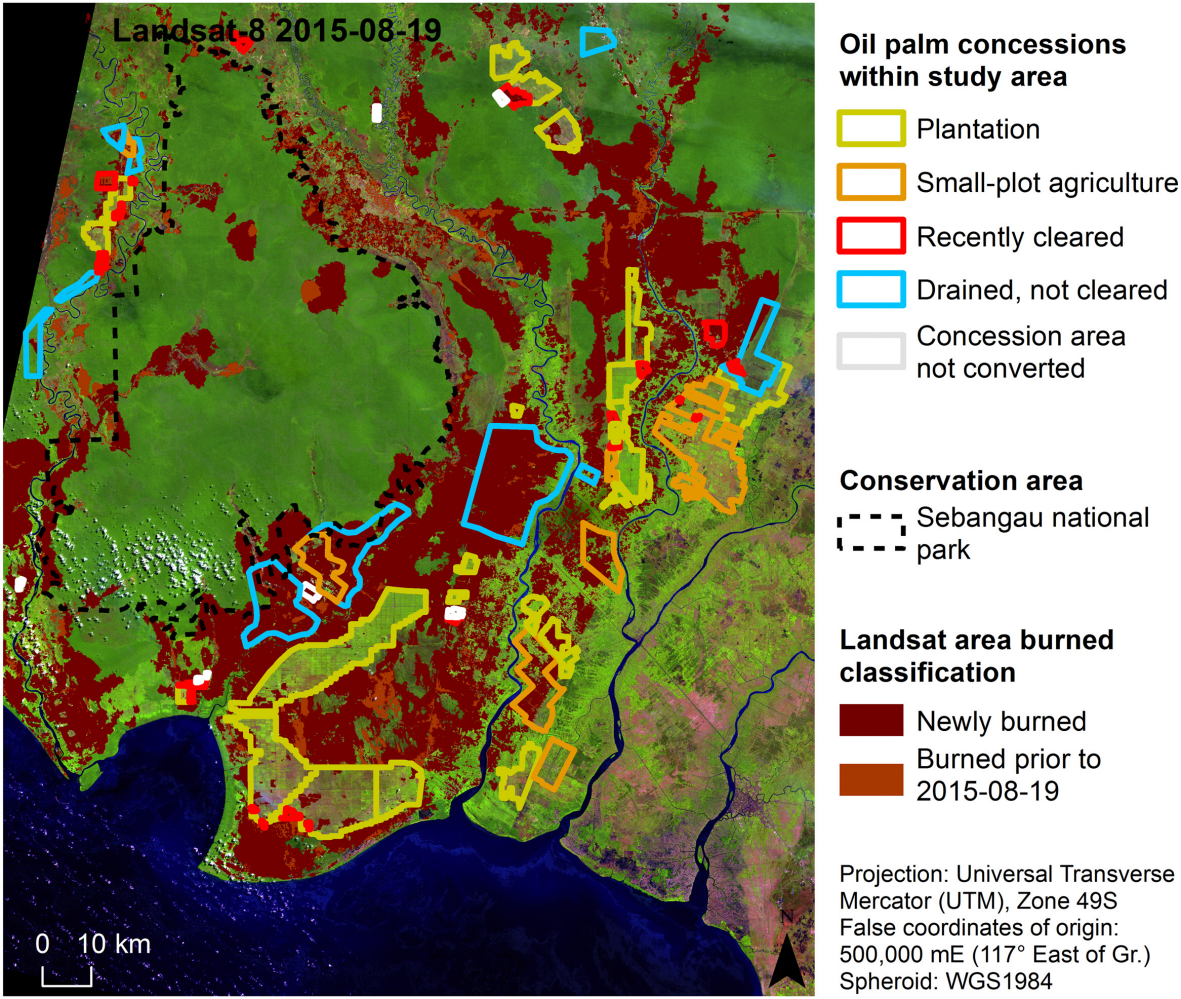
Oil palm plantation concessions and conservation areas. Landsat-8 OLI image (false color: R, band 9 1.36–1.38 μm; G, band 5 0.85–0.88 μm; B, band 4 0.64–0.67 μm; source USGS/NASA) from Aug. 19^th^, 2015, overlain with oil palm plantation concession classification, Sebangau national park boundary and Landsat burned area classification.

Oil palm plantation concessions for the study area were made available by the MoEF and accessed from Global Forest Watch [46]. These data indicated which concessions either hold or are in the process of obtaining a Right to Cultivate license (Hak Guna Usaha, HGU). Further visual analysis of Landsat-8 OLI imagery revealed several new cultivation areas, which were incorporated into the current analysis (Fig 2). Plantations which were planted were grouped as “Plantation”. Areas designated as a concession but being used for small-holder agriculture were grouped as “Small-plot agriculture”. Concessions that showed a spectral signal of bare ground or recently burned, but not yet planted, were grouped as “Recently cleared”, while those with indications of plantation infrastructure (such as drainage canals) but were still primarily covered with forest or scrubland were grouped as “Drained, not cleared”. Lastly concessions that were provided in the MoEF dataset but, based on Landsat imagery, did not appear to have any oil palm plantation infrastructure were grouped as “Concession area not converted”.

### Remote sensing systems

TET-1 is based on an improved version of the BIRD (Bi-spectral Infrared Remote Detection) satellite structure and was launched July 2012, which together with the BIROS (Berlin InfraRed Optical System, launched June 2016) satellite will comprise the DLR FireBird mission. In addition to offering a testing platform for space technology through the DLR On-Orbit Verification program, TET-1 strives to provide better detection capabilities to quantitatively analyze HTEs such as active fires and volcanoes. TET-1 is a microsatellite, measuring 65 x 55 x 88 cm in size and weighing 120 kg, in a Low Earth Orbit (LEO) at circa 500 km altitude. Its orbit time is 90 minutes, translating to a revisit time of maximum 5 days at latitude 40° north, although given the satellite’s off-nadir tilt and depending on location a target could be acquired on two consecutive days. The satellite is equipped with pushbroom sensors in the TIR (8.5–9.3 μm) and MWIR (3.4–4.2 μm), as well as three additional sensors in the VNIR (0.46–0.56 μm, 0.56–0.72 μm, 0.79–0.93 μm). Image swath width is 162 km for the TIR/MWIR bands and 202 km for the VNIR bands, and the ground sampling distance (GSD), or image pixel resolution, is on average 160 m. The onboard sensors not only enable the use of a bi-spectral method to provide subpixel fire radiative power estimates, the refined sensor sensitivity and a fine spatial resolution allow for improved detection and monitoring of a wider range of fires [43,48].

**Fig 3:**
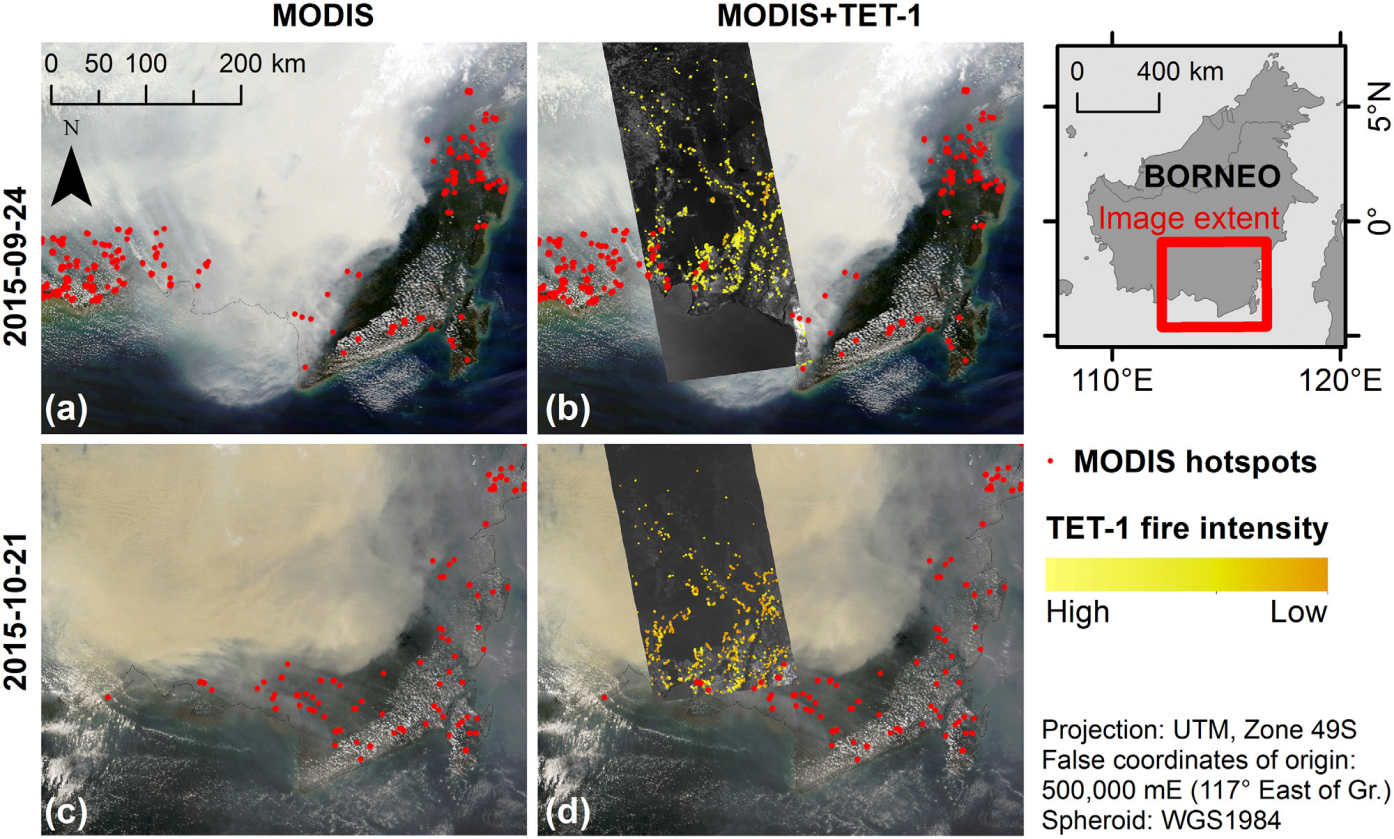
MODIS imagery and hotspot data compared with TET-1 imagery overlay. (a) MODIS Aqua true color (R, band 1 0.62–0.67 μm; G, band 4 0.55–0.57 μm; B, band 3 0.46–0.48 μm; source NASA) image from Sept. 24^th^, 2015, superimposed with same day MODIS hotspot data (red dots; source FIRMS collection MCD14). (b) The MODIS image overlaid with same day TET-1 gray-scale acquisition (source DLR FireBird Mission). MODIS hotspot data appear to under-detect low intensity fire fronts visible in TET-1 imagery (intensity of detected fire pixels indicated by yellow gradient). (c) MODIS imagery from Oct. 21^st^, 2015, superimposed with same day MODIS hotspot data. (d) The MODIS image overlaid with TET-1 imagery, which shows MODIS hotspot active fire detection being inhibited by thick smoke and haze.

The MODIS (Moderate Resolution Imaging Spectroradiometer) instrument, onboard both the Terra and Aqua satellites, provides multispectral detection capabilities comprising 18 bands in the VNIR (0.41–1.38 μm), 10 bands in the SWIR/MWIR (1.64–7.32 μm) and 8 bands in the TIR (8.55–14.23 μm). The satellites are orbiting at 705 km, with Terra imaging at 10:30 am on its descending node and Aqua imaging at 1:30 pm on its ascending node. Each platform delivers daily coverage of the entire globe. Data are provided at spatial resolutions of 250 m (2 bands), 500 m (5 bands), and 1 km (29 bands). Since their launch in 1999 and 2002, both satellites have proved a valuable resource for monitoring the atmosphere [49], land cover [50,51], vegetation [52], snow coverage [53], sea ice [54,55], sea surface temperature [56,57], and ocean color [58–60]. The MODIS Active Fire Product [42] and Burned Area Product [61,62] have both been extensively used to monitor fire occurrence worldwide [12]. Most bands saturate at brightness temperatures of 330–400 K, with the exception of the 3.9 μm low-gain band (channel 21) which saturates at 500 K. Quantification of very large fire events is hindered by a low sensor saturation temperature [43], and while MODIS has until now offered the highest sensor saturation range, the 1 km pixel resolution still limits detection capabilities of small fires and fire dynamics. The Burned Area Product has known issues detecting fire activity in Central Kalimantan [63], resulting from the algorithm being based on a 16-day cloud-free mosaic which is difficult to obtain in the tropics [61]. The MODIS Burned Area Product was therefore not considered in this study. An initial comparison of the MODIS Active Fire Product (hotspots collection MCD14) and TET-1 imagery suggests that TET-1 can provide improved detection of small fire fronts (Fig 3a and 3b) as well as better signal detection through thick smoke and haze (Fig 3c and 3d).

The Landsat mission provides one of the longest continuous global imaging records available, covering from 1972 until present, and now delivers multispectral images in the VNIR, SWIR and TIR at a spatial resolution of 30 m. The current operational constellation consists of Landsat-7 ETM+ and Landsat-8 OLI, which when taken together provide a revisit time of 8 days. It should be noted that the Landsat-7 ETM+ dataset is reduced due to the Scan Line Corrector failure since 2003, which results in gaps of no data within images. Landsat satellite imagery has been used extensively for detecting area burned following a fire event, but such analyses can be hampered by heavy cloud and haze cover [34,64]. The recently launched European Space Agency (ESA) Sentinel-2A mission in June 2015 also provides fine resolution (down to 10 m), multispectral imagery in the VNIR and SWIR, and is expected to enable high quality analyses of land cover [65]. During the study period, Sentinel-2A provided a revisit time of 10 days over the study area. Both Landsat and Sentinel-2 images were considered to estimate change in burned area prior to and following the fire event. An overview of the all remote sensing datasets analyzed is provided in Table 1.

**Table 1:** Overview of Remote Sensing Image Data.

Sensor	Acquisition date	Image ID
**TET-1**	2015-09-24	FBI_TET1_20150924T051034_20150924T051134_L1B_C_EL
	2015-10-05	FBI_TET1_20151005T050624_20151005T050738_L1B_C_MH
	2015-10-13	FBI_TET1_20151013T051039_20151013T051153_L1B_C_EL
	2015-10-15	FBI_TET1_20151015T171847_20151015T171956_L1B_C_EL
	2015-10-18	FBI_TET1_20151018T170832_20151018T170941_L1B_C_EL
	2015-10-21	FBI_TET1_20151021T051425_20151021T051549_L1B_C_EL
	2015-10-23	FBI_TET1_20151023T172140_20151023T172259_L1B_C_EL
**MODIS hotspots**	2015-06-01 to 2015-12-31	MCD14
**Landsat**	2015-08-19	LC81180622015231LGN00
	2015-11-23	LC81180622015327LGN00
	2015-12-01	LE71180622015335EDC00
	2015-12-09	LC81180622015343LGN00
**Sentinel-2A**	2015-12-23	S2A_OPER_MSI_L1C_TL_SGS_20151223T061706
	2015-12-26	S2A_OPER_MSI_L1C_TL_SGS_20151226T094222

### Satellite image processing

Taking into account both day and nighttime images, the TET-1 revisit time over the study area is between 2 and 3 days. At-sensor radiance images falling within the study area during the period of interest were supplied by the DLR Institute of Optical Sensor Systems in Berlin. Only the MWIR band was utilized in this study. Images did not always cover the full spatial extent of the study area. Further post-processing of images included image georeferencing to a Universal Transverse Mercator (UTM), zone 49 South, projection using ENVI 5.0 (Exelis Visual Information Solutions GmbH) and subsequent additional geocorrection in ArcMap 10.2.2 (ESRI Inc.).

MODIS hotspot data (MCD14) were accessed through the Fire Information for Resource Management System (FIRMS). To compare MODIS and FireBird sensor systems, hotspot data were post-processed to overlap temporally with the TET-1 imagery data, meaning data from days without TET-1 images were removed. Since on a given day TET-1 imagery did not always extend over the entire study area, the MODIS hotspot dataset was further clipped to ensure identical spatial coverage from both sensor datasets.

Relatively cloud and haze free (< 60% cloud-coverage) Landsat images over the study area from the period June 2015 through January 2016 were accessed from the U.S. Geological Survey (USGS) GloVis server. Images from the Sentinel-2 mission were also considered, but only images with heavy cloud-coverage (> 60%) over the study area from two months post fire event (November and December) were available and thus only used to qualitatively evaluate the Landsat burned area product. Both Landsat and Sentinel-2A images were atmospherically corrected using ATCOR-2/3 software (developed by Dr. Rudolf Richter, now licensed by ReSe Applications Schläpfer) [66].

### Classification of active fires and burned areas

Hierarchical object-based image analysis (OBIA) is a recently developed technique that evaluates spectral band information combined with spatial context and pattern recognition algorithms [67]. This approach has been found to outperform traditional pixel-based classification methods when working with fine spatial resolution remote sensing imagery [68,69]. TET-1 and Landsat images were classified with eCognition software (Trimble Navigation Ltd.) using a hierarchical OBIA approach. To classify active fire pixels from TET-1 MWIR images, a ruleset was developed based on image-specific object values such as scene mean and standard deviation, mean difference to neighbors, abrupt boundary transition values and proximity to very bright objects. Water reflection pixels were removed based on a river+ocean mask produced from OpenStreetMaps (access date: Nov. 17^th^, 2015). An accuracy assessment was performed comparing the hierarchical OBIA results with a separate evaluation conducted by an independent analyzer. The assessment was based on a stratified random sampling scheme to control for the much lower coverage of fire pixels within an image, where 100 points were randomly assigned within each category (fire and non-fire, making a total of 200 assessment points per image), and an adjusted (weighted) error matrix was calculated based on area-normalized proportions [70,71].

Landsat images were used to classify recently burned areas both prior to and following the TET-1 imagery time series. A Landsat-8 OLI image from August 19^th^, 2015, with only 2% cloud-coverage, was classified using an OBIA ruleset based on the Normalized Burn Ratio (NBR) and the product of a spectral unmixing analysis for recently burned areas, similar to methods developed by Hoscilo et al. [34] and Hoscilo et al. [72]. No clear images were available from November 2015 to January 2016 (46–58% cloud-coverage), and analysis was therefore limited to cloud and haze free areas within the image. Classification of recently burned areas from two separate Landsat-8 OLI images (Nov. 23^rd^ and Dec. 9^th^, 2015) plus one Landsat-7 ETM+image (Dec. 1^st^, 2015) were combined to create a coverage estimate of recently burned areas. Areas of no data due to cloud coverage in all three images but clearly within a burn area, defined as being completely enclosed, were included in the post fire event classification. Despite these efforts, this method is likely underestimating the amount of recently burned area, spotlighting a limitation of Landsat imagery in capturing recently burned areas due to being dependent on cloud and haze free conditions.

### Comparison of TET-1, MODIS hotspots and Landsat imagery

Active fire detection capabilities were compared between TET-1 imagery and MODIS hotspot data. Both datasets were spatially clipped to include the study area, and only hotspot data taken on days with TET-1 acquisitions were used. As mentioned, TET-1 imagery did not always completely cover the study area on a given day, which was controlled for in the MODIS hotspot data by subsetting both spatially and temporally.

The Landsat recently burned area classification was compared to a MODIS hotspot burned area estimate. Each MODIS hotspot was assumed to represent a square 1-km pixel. Downloaded MODIS hotspot data were reduced to overlap the same time period covering all Landsat images (2015-08-19 to 2015-12-09), then spatially clipped to the study area and dissolved to remove overlapping pixel areas.

### AOI (Areas of Interest) selection and measuring fire front propagation speed

Fire AOI’s were selected for further analysis dependent upon existence of a sufficient time series, defined as a series of active fire pixels from the TET-1 imagery covering at least three separate dates (see Fig 1). Areas were considered both within the Sebangau National Park as well as in neighboring degraded regions and oil palm plantation concessions. For each fire AOI, fire front propagation speed was measured by comparing the location of a fire front from one date to the next. Fire front propagation lines were placed so that they lay as perpendicular as possible to the advancing fire line and all notable fire propagation directions were being assessed. To obtain fire propagation speeds, the distance between fire fronts from one date to the next was divided by the number of intervening days.

Area burned within each fire AOI was quantified from the digitized TET-1 classification results as well as the Landsat classification results. When ground types were different within an AOI, the AOI was classified using the predominant ground (or vegetation) type covering the area. Within the AOI, each fire propagation line was classified based on the ground (or vegetation) type lying below the vector’s middle point.

## Results

### TET-1 classification and comparison with other sensors

The accuracy assessment of the OBIA active fire analysis was found to have an adjusted overall accuracy of 93% or higher for each TET-1 image. In comparing active fire detection capabilities of TET-1 and MODIS (Table 2), the TET-1 data were clearly outperforming the hotspots data. During the time period of Sept. 24^th^ to Oct. 23^rd^, the MODIS hotspot algorithm detected 1,090 active fire pixels when controlling for consistent spatial extent. This translates to an active fire area estimation of 109,000 ha. During the same time period, TET-1 detected 88,704 active fire pixels which translate to an active fire area estimation of 225,469.44 ha.

**Table 2:** Comparison MODIS and TET-1 Active Fire Detection.

Sensor	Revisit time (days)	Spatial resolution (m)	Detected active fire pixels	Estimated area (ha)	Percent study area
**MODIS hotspots**	0.5	1,000 x 1,000	1,090	109,000.00	4.5%
**TET-1**	2–3	160 x 160^a^	88,074	225,469.44	9.3%

^a^Ground resolution varied from 148 m to 169 m between images.

The Landsat recently burned area OBIA analysis, comparing changes from 2015-08-19 to 2015-12-09, resulted in a burned area estimate of 684,561.47 ha, while the MODIS hotspot algorithm, when controlling for concurrent spatial and temporal coverage, detected 13,225 active fire pixels and after being dissolved translated to an estimated burned area of 496,124.68 ha (Table 3). As mentioned previously, the Landsat images from November/December 2015 had between 40–60% cloud-coverage, which created areas of “No data due to cloud cover” within the final classification. Areas which were clearly enclosed by burned areas were included in the Landsat burned area estimate, amounting to 22,314.81 ha or 3.3% of the total estimate.

**Table 3:** Comparison MODIS and Landsat Burned Area Detection Capabilities.

Sensor	Revisit time (days)	Spatial resolution (m)	Detected burned area pixels	Detected active fire pixels	Estimated burned area (ha)	Percent study area burned
**MODIS hotspots**^a^	0.5	1,000 x 1,000	N/A	13,225	496,124.68	20.4%
**Landsat**^b^	8	30 x 30	7,606,239	N/A	684,561.47	28.2%

N/A, detection method not applicable to the dataset.

^a^MODIS hotspot data cover the same period of time as the before and after Landsat images.

^b^Analysis based on comparison of detected burnt area from pre-fire (2015-08-19) to post-fire (2015-10-23, 12–01 and 12–09) images.

### Fire front analysis and area burned within each AOI

**Fig 4:**
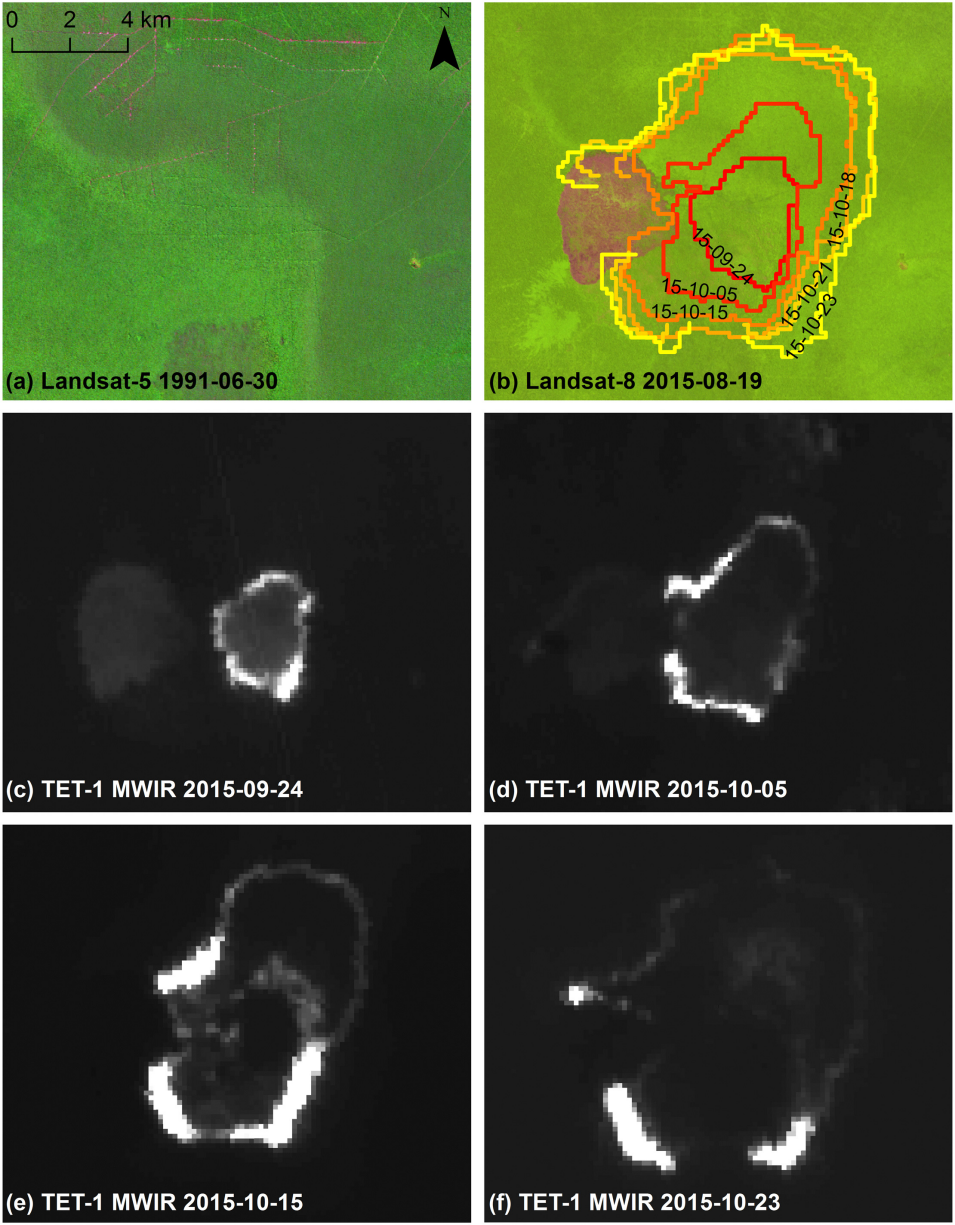
Symmetric ring fire front (F03) time series. (a) Landsat-5 TM imagery (false color: R, band 5 1.55–1.75 μm; B, band 4 0.76–0.90 μm; G, band 3 0.63–0.69 μm; source USGS/NASA) from June 30^th^, 1991, showing historical logging railway infrastructure and burn scars (purplish region) along southern image edge. (b) Landsat-8 OLI imagery (false color: R, band 9 1.36–1.38 μm; G, band 5 0.85–0.88 μm; B, band 4 0.64–0.67 μm; source USGS/NASA) from Aug. 19^th^, 2015, overlain with TET-1 detected fire front time series from six acquisition dates. Recently burned area prior to fire event is located along the western fire edge. Original TET-1 midwave infrared (MWIR; source DLR FireBird Mission) imagery is shown for (c) Sept. 24^th^, (d) Oct. 5^th^, (e) Oct. 15^th^, and (f) Oct. 23^rd^, 2015.

**Fig 5:**
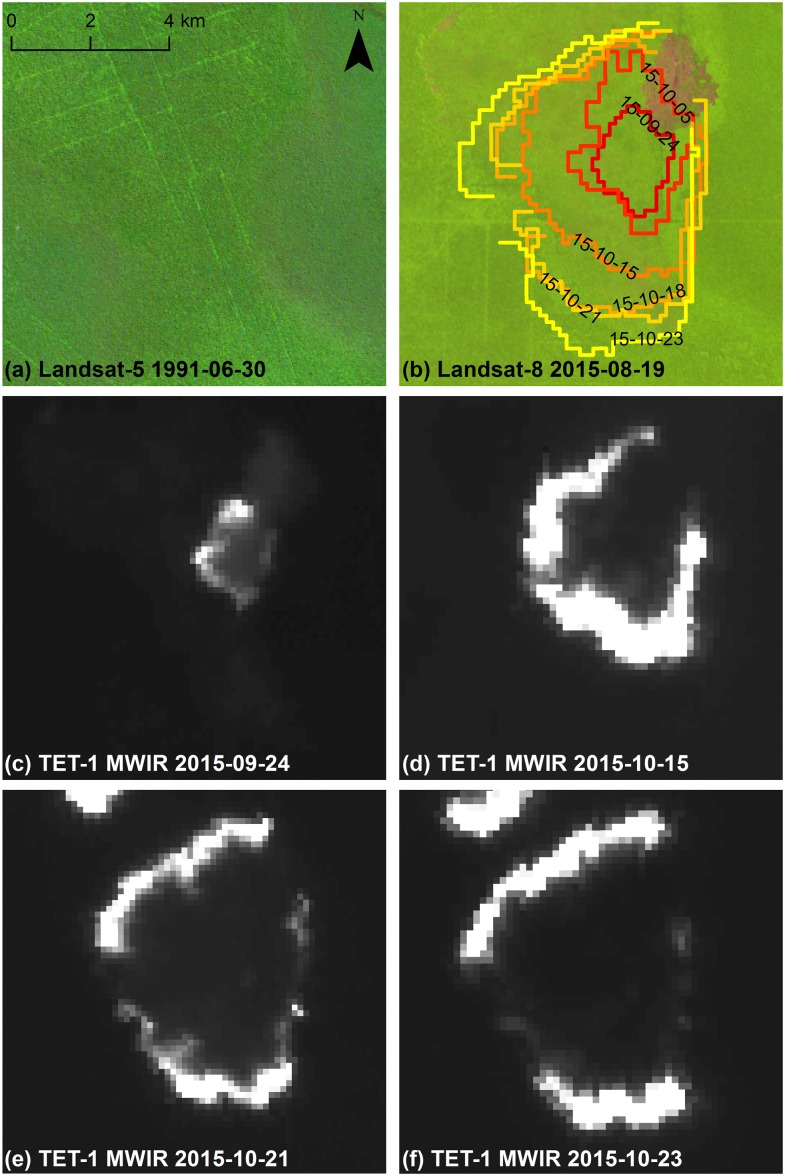
Asymmetric ring fire front (F02) time series. (a) Landsat-5 TM imagery (false color: R, band 5 1.55–1.75 μm; B, band 4 0.76–0.90 μm; G, band 3 0.63–0.69 μm; source USGS/NASA) from June 30^th^, 1991, showing historical logging railway infrastructure. (b) Landsat-8 OLI imagery (false color: R, band 9 1.36–1.38 μm; G, band 5 0.85–0.88 μm; B, band 4 0.64–0.67 μm; source USGS/NASA) from Aug. 19^th^, 2015, overlain with TET-1 detected fire front time series from six acquisition dates. Recently burned area prior to fire event is located along the northeastern fire edge. Original TET-1 midwave infrared (MWIR; source DLR FireBird Mission) imagery is shown for (c) Sept. 24^th^, (d) Oct. 15^th^, (e) Oct. 21^st^, and (f) Oct. 23^rd^, 2015.

Comparing TET-1 active fire pixels over successive dates revealed interesting differences in fire propagation dynamics. Ring fires were found to be either symmetric (Fig 4) or asymmetric (Fig 5). In both figures, the outer fire front from each TET-1 image within the time series is displayed as a colored fire isochrome with the respective image acquisition date indicated. Oftentimes fire ring propagation would begin by spreading symmetrically in all directions only to encounter areas where fire propagation would be either slowed or even remain stationary. Previous fire scars (pink/purplish areas) and changes in logging infrastructure (rails and canals) are evident from the Landsat imagery from 1991 and 2015 (Figs 4a, 4b and 5a, 5b). The TET-1 MWIR data from different points within the time series are also presented (Figs 4c–4f and 5c–5f). It is evident from both figures that fire propagation speed over previous fire scar areas is either greatly slowed or the fire becomes no longer detectable. Other reasons for reductions in fire propagation speeds, such as observed along the southern border of the Fig 4 fire or the eastern border of the Fig 5 fire, are likely due to differences in vegetation or ground type and are discussed in more detail below.

Fire propagation speed descriptive statistics for each AOI, along with active fire area estimates from TET-1 images and burned area estimates from Landsat images, are presented in Table 4. The highest fire propagation speeds measured were in excess of 500 m/day. One can see from the standard deviation and the spread between maximum to minimum measurements that fire propagation speed was highly variable over all areas. This variability is likely partially due to wind patterns and fuel load, both of which could not be integrated into this study. The most common fire form observed was a long fire line discerned to have started from either a single (6 AOIs in total) or multiple points (7 AOIs in total), the second most common form was a fire ring spreading from a central location (the remaining 3 AOIs). The predominant ground and vegetation type for each AOI is also indicated. The three highest averages (Area ID F07, F14, F16) as well as the three highest maximum fire propagation speeds (F07, F09, F14) were all measured over peat greater than 2 m deep. The three areas found to have burned the most (F06, F09, F14) all resulted from fire lines that appeared to have been started from multiple sources.

**Table 4:** Propagation Speed and Fire Dynamic Measurements.

Area ID	N	Fire form	Ground type	Vegetation type	Fire propagation speed (m/day)				TET-1 active fire area (ha)	Landsat burnt area (ha)
					Average	S.D.	Minimum	Maximum		
F01	3	Line P	peat 1–2 m	MS	141.17	122.86	4.09	442.67	860	1,305
F02	9	Ring AS	peat >2m	TI+LP	128.82	112.30	5.63	514.50	3,044	3,914
F03	12	Ring SY	peat >2m	TI+LP	166.35	141.03	26.91	765.50	5,093	6,415
F04	6	Ring SY	peat >2m	LP+MS	172.28	126.93	7.00	388.67	925	990
F05	7	Line M	peat+sand	LP+MS	128.96	134.23	4.62	490.00	3,309	9,308
F06	8	Line M	peat >2m	LP+MS	163.92	167.26	10.60	854.50	5,827	9,948
F07	8	Line M	peat >2m	LP+MS	223.16	327.32	35.89	1696.00	4,295	9,478
F08	5	Line P	peat+sand	LP+MS	141.79	125.03	29.62	488.50	1,632	3,447
F09	8	Line M	peat >2m	LP	191.85	257.57	16.52	1054.50	5,425	15,992
F10	4	Line P	peat 1–2 m	LP+MS	87.94	43.17	45.80	175.67	659	1,790
F11	4	Line P	peat 1–2 m	LP+MS	143.69	107.68	12.79	360.00	1,431	2,150
F12	4	Line P	peat+sand	LP+MS	204.81	229.67	17.33	978.50	2,027	2,931
F13	5	Line P	peat 1–2 m	LP+MS	191.64	178.20	38.63	533.00	1,738	5,611
F14	15	Line M	peat 1–2 m and >2m	LP+MS	207.50	330.64	8.14	1903.50	7,502	23,367
F15	4	Line M	peat >2m	C	139.59	166.14	5.21	503.50	1,961	4,070
F16	5	Line M	peat >2m	C	258.54	233.82	46.63	828.50	1,447	4,456

N, number of fire propagation time series lines measured per AOI; S.D., standard deviation; Line M, line fire front started from multiple sources; Line P, line fire front started from a point source; Ring SY, symmetrical ring fire form; Ring AS, asymmetrical ring fire form; MS, mixed swamp forest; LP, low pole forest; TI, tall interior forest; C, concession.

### Fire propagation speed over different ground and vegetation types

Descriptive statistics showing the comparison of fire propagation speeds over different ground types is presented in Table 5. Differences in fire propagation speed between different ground types were not found to be statistically different (Mann-Whitney U-test or Wilcoxon Ranked-Sum Test, p>0.05), although certain trends can be observed. The highest average and maximum fire propagation speeds occurred over peat greater than 2 m deep. Average fire propagation speed reduces successively as the peat layer depth decreases to a thin peat layer lying over sand.

**Table 5:** Fire Propagation Speeds over Peat and Sand Ground Types.

Ground type	Propagation speed (m/day)				
	N	Average	S.D.	Minimum	Maximum
peat+sand (depth unknown)	14	97.63	87.62	20.50	361.00
peat 0–1 m	30	134.94	131.90	4.62	490.00
peat 1–2 m	77	161.53	172.53	4.09	978.50
peat > 2m	202	187.01	237.82	5.21	1903.50

N, number of fire line measurements; S.D., standard deviation.

Fire propagation speeds over various vegetation types is presented in Table 6. Differences were only tested for the vegetation classes which contained more than 10 fire line measurements (secondary swamp forest and swamp scrubland), but no significant differences were found (Mann-Whitney U-test or Wilcoxon Ranked-Sum test, p>0.05). The slowest propagation speeds were observed in the planted plantation class. Although the quickest propagation speed was observed over the swamp land cover class, it should be noted that this class is represented by only a single measurement. The highest maximum propagation speed (1,903.50 m/day) was observed over previously unburned secondary swamp forest.

**Table 6:** Fire Propagation Speeds over Different Vegetation Types.

Vegetation type	Propagation speed (m/day)				
	N	Average	S.D.	Minimum	Maximum
Planted plantation	5	125.99	146.43	14.76	415.00
Secondary swamp forest	199	185.72	243.34	4.09	1903.50
Swamp scrubland	118	151.12	145.46	5.21	828.50
Swamp (grass & sedge)	1	207.33	N/A	N/A	N/A

N, number of fire line measurements; S.D., standard deviation.

### Fire prevalence in relation to different levels of concession usage

Occurrence of fire under different levels of concession usage is presented in Table 7. The two largest usage categories by area were “Plantation” and “Concession area not converted”, and both these areas were also found to contain the highest active fire area estimates (5,297 ha and 1,717 ha respectively). By normalizing area coverage, accomplished by dividing the active fire area with the total area within a concession category, one observes that fires occurred most frequently in concession areas that are “Drained, not cleared” (13.5% within the area, 8.7% along the border, and 32.7% within 500 m of the border). Fires were found to occur least often in the small-holder agricultural areas (3.2%), and plantation areas (4.1%).

**Table 7:** Oil Palm Plantation Fire Occurrence.

Current status	Total parcels	Area (ha)	Number with HGU	TET-1 active fires (ha; % by area)		
				Inside	Border 160m	Within 500 m
Plantation	25	129,140	7	5,297; 4.1%	1,309; 1.0%	4,246; 3.3%
Small-plot agriculture	11	54,435	1^a^	1,717; 3.2%	82; 0.2%	300; 0.6%
Recently cleared	19	8,606	3	468; 5.4%	188; 2.2%	573; 6.7%
Drained, not cleared	7	1,994	0	270; 13.5%	173; 8.7%	652; 32.7%
Concession area not converted	10	77,703	1 ^a^	6,459; 8.3%	456; 0.6%	1,544; 2.0%

HGU, Cultivation Right on Land (Indonesian: Hak Guna Usaha).

^a^Plots granted with only local permits.

## Discussion

Over the study period, TET-1 detection of active fire pixels consistently outperformed the MODIS hotspot algorithm. Even when accounting for differences in image pixel resolution (1 MODIS pixel is equivalent to circa 39 TET-1 pixels), the MODIS hotspot data detected less than half the active fires as compared with TET-1. These results are consistent with findings in other studies [43]. The MODIS hotspots burned area estimate, based on hotspot active fire detections converted to burned area, was also outperformed by the Landsat OBIA burned area analysis, which estimated 38% more newly burned area despite the data being of lower quality due to haze and cloud cover. The assumption that a MODIS hotspot point represents a complete square kilometer of burned area is tenuous and likely presents an overestimation of burned area detection. This only further supports the conclusion that Landsat should be the preferred passive detection system for burned area estimates following a fire event, however analyses of fire dynamics are very limited with this sensor given revisit time and cloud, haze and smoke coverage. Potential issues presented by comparing an algorithm detecting a dynamic process (such as active fire) with an algorithm detecting the product of a process (burned area) are discussed below. Fire detection issues with haze and cloud cover for both Landsat as well as MODIS are not unknown [34,64], and Fig 3 displays excellent examples of thick haze hampering the ability of the MODIS sensor to detect active fires. While MODIS, with a high saturation temperature of 500 K in one of the MWIR bands and global coverage every day, has been and continues to be the workhorse of global fire detection, the coarse 1-km spatial resolution detracts from the sensor’s capability to capture small fire events and fronts [43]. The improved ability of the TET-1 sensor to capture these dynamics is displayed well in Fig 3b and 3d, where multiple smaller fire fronts are detected which were not present in the MODIS hotspot data.

TET-1 was intended primarily as an experimental satellite platform, and as such this study was conducted under certain limitations. An atmospheric correction of the MWIR band was not possible using ATCOR (pers. comm. R. Richter). Radiance in the MWIR spectrum is primarily affected by aerosols and water vapor in the atmosphere, and working with non-atmospherically corrected data most likely increases issues with false positive detection. We controlled for issues with sun glint from water and bright land cover types, such as bare soil with high quartz sand content, through utilization of a water mask and focusing analyses on TET-1 image time series that displayed similar patterns over 3 separate dates. Synergy of atmospherically corrected TIR and MWIR band data would enable better quantification of characteristics such as fire radiative power [43], and further research is currently being conducted to address the need for an appropriate atmospheric correction for data from this sensor. TET-1 has been joined in 2016 by BIROS, which will lower time between acquisition dates and provide an opportunity to reduce false positive detections through image comparison. Additionally, an operational bi-spectral method product based on the MWIR and TIR bands is in development, which will enable subpixel analysis of fire temperature and area. A measurement of fire temperature, an indicator of fire intensity, could enable earlier estimation of fire emissions from a particular area.

Derivation of burnt area estimates from active fire detection algorithms have been found to be prone to error [31], primarily due to available active fire detection systems producing only a snapshot of a continuously moving fire front. Our estimates of actively burning area from TET-1 (225,469.44 ha) were not surprisingly much less than those from the burned area Landsat analysis (684,561.47 ha). Under the current FireBird satellite constellation, TET-1 acquisitions were only possible every 2–3 days. We often observed jumps in the detected fire pixels from one image to the next, which were assumed to be due to quickly spreading fire within that period of time. A similar trend appeared when comparing the AOI active-burning area detected by TET-1 with the newly burned area detected by Landsat (Table 4), where TET-1 was found to be underestimating the area by up to 69%. The two area estimation methods came the closest to one another for the F04 fire (925 ha and 990 ha respectively), which could be expected since it was a relatively small fire with few large jumps observed in the TET-1 time series. This issue will be lessened by expanding the FireBird constellation and thus shortening the period of time between image acquisitions, but it should be noted that products from algorithms for detecting active fires should be expected to be fundamentally different than algorithms for detecting burned area after a fire event.

Measured fire propagation speeds were highly variable both between and within different fire AOI’s (Table 4), and the highest propagation speeds observed were in excess of 500 m/day. Average fire propagation speeds measured are on par with those estimated for cleared tropical rainforest (202 m/day) [73], but are much higher than estimates by Usup et al. [22] for peat fires in the same region (0.3–0.9 m/day). Distributions of the fire propagation speeds were highly skewed, as indicated by the median being often smaller than the arithmetic average, but even when considering only the median, a two orders of magnitude reduction in the propagation speed was not revealed. Possible causes for the propagation speed discrepancy could be the study by Usup et al. [22] being conducted during a less severe ENSO event, thus under different peat moisture content conditions, and over a different vegetation type than present in many AOI’s analyzed in this study. The observed fire ring forms resemble those predicted by Usup et al. [22], where surface peat fire fronts move in an erratic pattern determined by distribution of favorable ignition conditions and can burn into deeper peat layers. Fire fronts were found to slow or even stop when encountering an area that had been recently burned (excellent examples can be observed around the pink/purple areas in Figs 4b and 5b). This is not surprising as aboveground biomass is reduced through fire, with tropical forest taking many decades to recover [74], thus the observed slowed propagation speed could be due to lower fuel availability.

No significant difference was found in fire propagation speed over unburned secondary swamp forest and swamp scrubland that burned sometime within the last 30 years. The MoEF land cover classification is based on 30 m x 30 m resolution Landsat data but created using a manual delination approach that utilized a minimum mapping unit of 6.25 ha [44,75,76]. TET-1 pixels correspond to an area of 2.56 ha, thus the two datasets are within the same order of magnitude of one another. Despite this, these data are likely not detailed enough to sufficiently capture the relationship between different fuel loads available in various forest types such as tall interior, low pole and mixed swamp forest. Fire propagation lines were observed to slow when moving from tall interior peat swamp forest to low pole or mixed swamp forest. These forest types are evident in Fig 5a, where logging railways together with the bright-green textured area in the image center indicate tall interior forest containing valuable timber species. To the East of this area, low pole/mixed swamp forest is indicated through very little logging infrastructure and less green textured area. In Fig 5b, a seeming fire propagation boundary occurs along the same area as this forest type boundary. This can also be observed in Fig 4b along the northwestern and eastern edges of the F03 fire ring. The northern edge of this fire burned into an area appearing to be tall interior forest (logging infrastructure), but despite this the fire front quickly slows after 2 weeks of burning. Low pixel brightness temperature, a proxy for fire intensity, along these edges can be observed in the original TET-1 images (Fig 4d–4f), and may be indicative that the fire is slowly spreading through the deeper peat layer. From the November/December Landsat images, this area appeared to still contain many patches of partial green, providing supporting evidence for a low intensity fire front. This, together with the fire front’s persistence for more than 2 weeks, suggests that the deep peat layer had begun to slowly burn, but without ground truth measurements, a conclusive determination is outside the scope of this study.

It is interesting to note in Table 5 that fires propagating over peat+sand were never found to burn slower than 20 m/day. The minimum propagation speeds in the other ground type categories (peat depths 0–1 m, 1–2 m, and > 2 m) were higher than those found by Usup et al. [22], although are closer to peat fire propagation speeds measured in Russia (2.4 m/day) [77] and Canada (2.9 m/day) [78]. Quickly spreading surface peat fires were likely mixed with deeper peat fires for each category during the analysis, which together with factors such as differing fuel availability and weather conditions, could explain the large variation in the data. The analysis was limited to what is possible to measure using a remote sensing system and there is a dire need to build upon the work of Usup et al. [22] in order to provide further *in situ* measurements of peat fire propagation speeds in Central Kalimantan over different ground and vegetation types.

**Fig 6:**
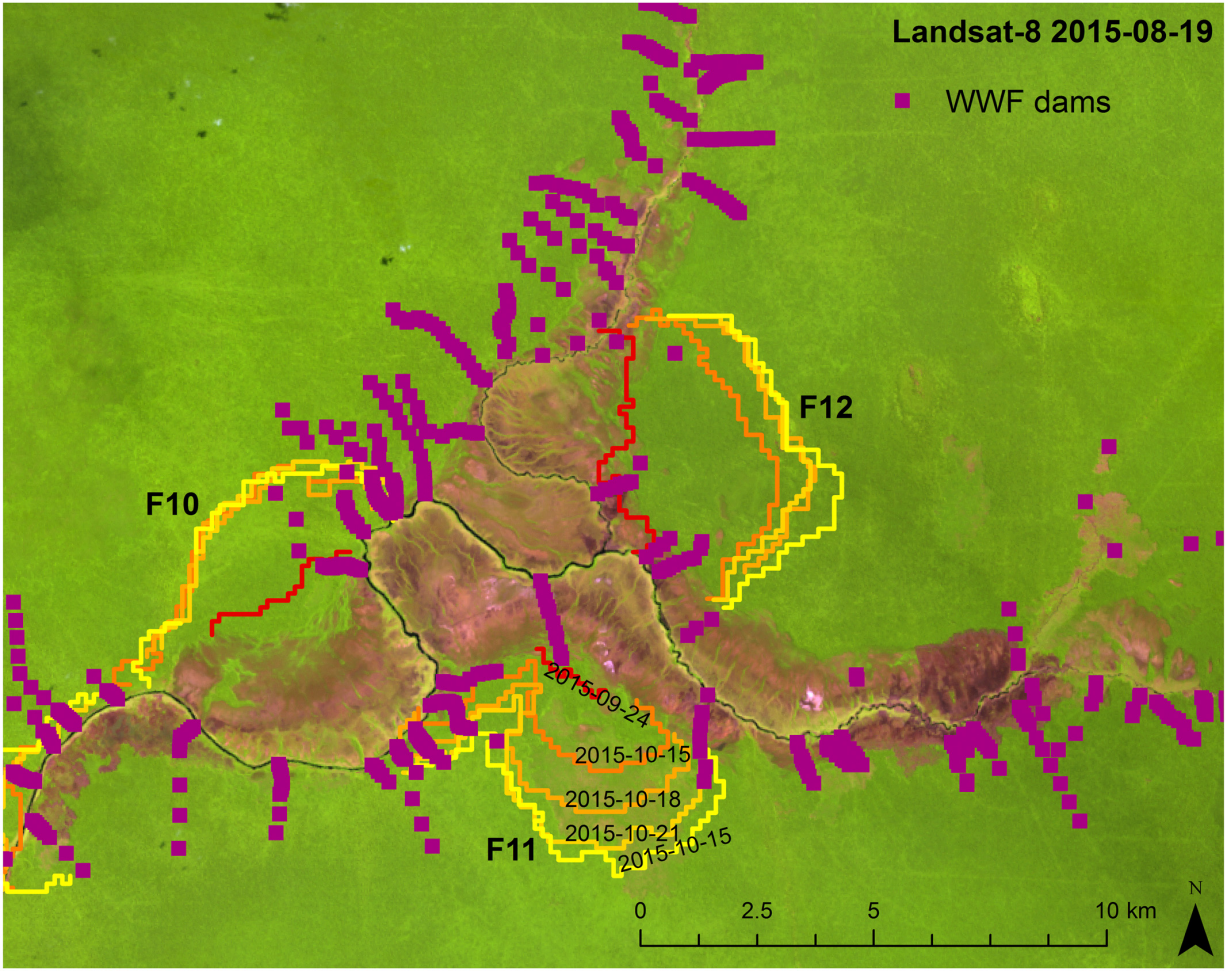
Fire front dynamics of AOI F10, F11 and F12 in relation to installed dams. Landsat-8 OLI imagery (false color: R, band 9 1.36–1.38 μm; G, band 5 0.85–0.88 μm; B, band 4 0.64–0.67 μm; source USGS/NASA) from Aug. 19^th^, 2015, overlain with TET-1 detected fire front time series from six acquisition dates. Location of dam installations shown by purple squares.

Another potentially important factor not included in the analyses was water table level, as peat with low moisture content has a much higher risk of catching on fire [22]. Conservation efforts within the Sebangau National Park have included installing dams to help slow run-off and thus retain more water in the peat swamp forest [45]. Whether these dams have a dampening effect on fire dynamics could be observed during the 2015 fires (Fig 6). Three different fire fronts were measured (F10, F11, and F12), one of which had the lowest average fire propagation speed measured (F10, 87.94 m/day, see Table 4). The F10 and F11 fires occurred over peat > 2m deep, and the slow propagation speed of the F10 fire is likely due to the deeper peat layer catching fire. The F11 fire appears to be a quick-moving surface peat fire front. Installed WWF dam locations are also presented in Fig 6. It can be observed that the East and West boundaries of the F10 and F11 fires correspond to dam installation locations, while the central portions of these fires contain little to no dams. The F12 fire occurred over thin peat covering a quartz sand layer (peat+sand). From the fire front time series, this fire appears to have begun as a quick-moving surface fire and then slowing (similar to the F10 fire). The centrally located dams for the F12 fire did not appear to have the same dampening effect observed for the F11 and F10 fire. This may be due to the shallowly located sand layer, which presents different groundwater porosity conditions than peat. The TET-1 data alone can simply offer qualitative observations on dam effectiveness, and conclusions should only be made after extensive *in situ* sampling, but the opportunity to use observed fire dynamics to focus successful field campaign efforts post-fire event can be highlighted by this example.

From the analysis of fire occurrence in different concession areas (Table 7), it is evident that fires occur most often in plots with installed drainage infrastructure but have not yet been cleared. Since fire is the technique of choice to quickly and cheaply clear slashed areas [27], the frequent occurrence of fire within the “Drained, not cleared” areas is not surprising. This concession class also had the highest percentage of fire occurrence along its borders and within 500 m thereof, supporting the hypothesis that most peat swamp fires originate from anthropogenic sources [18]. Interestingly enough, small-plot agriculture areas had some of the lowest percentages of fire occurrence. Fires were often first detected in previously burned areas which then spread into surrounding primary forest. Indonesia has a long history with oil palm plantation management [18], including actions to control concession growth through a moratorium upon issuing new licenses in 2011 [79]. Low-intensity peatland fires can contribute heavily to emissions [29,78,80], and reducing their occurrence as well as their size will play an important factor in Indonesia’s plan to reduce emissions 26% by 2020.

**Fig 7:**
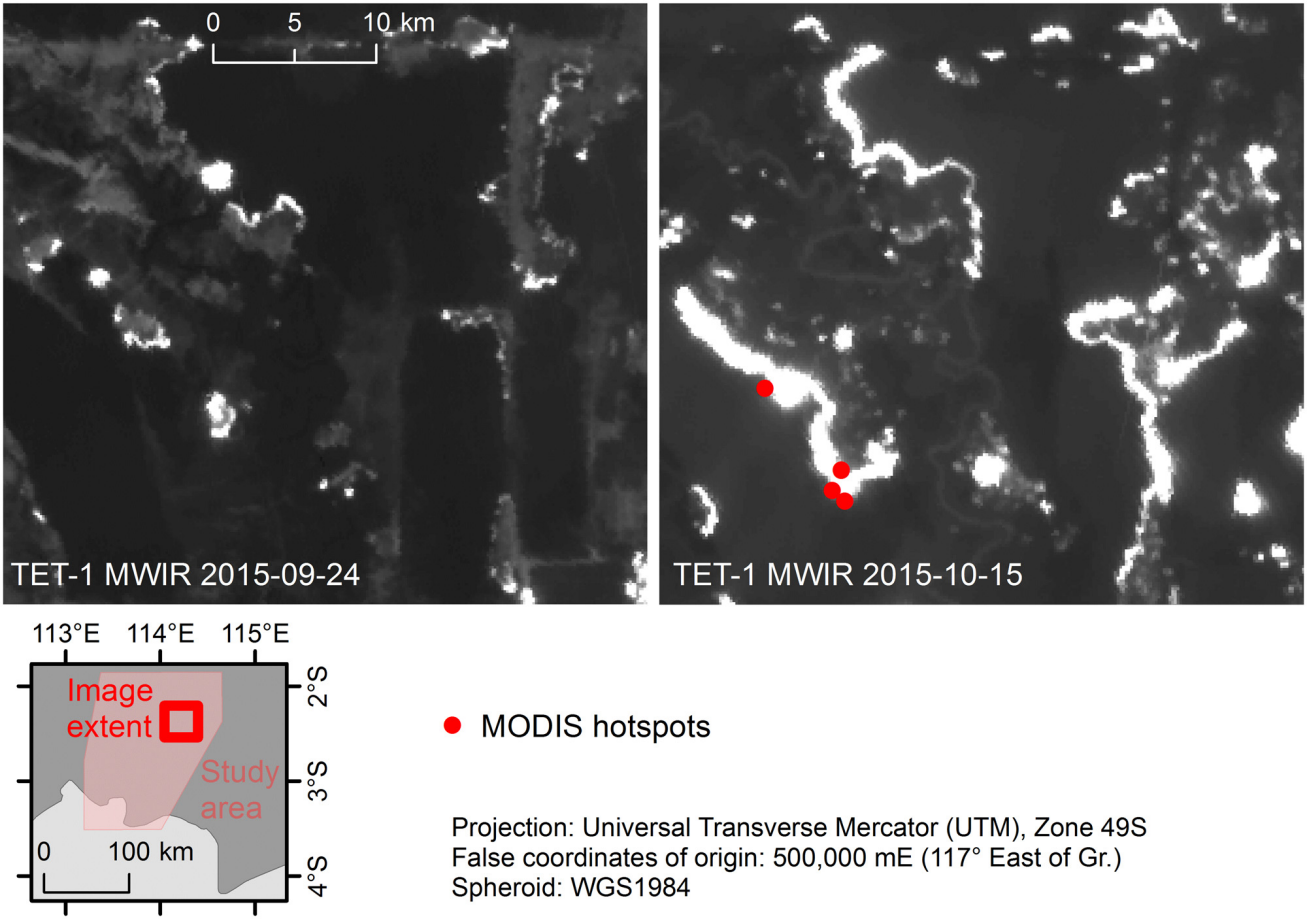
Rapid fire front growth during 2 weeks and comparing MODIS hotspot fire detection with TET-1. TET-1 midwave infrared (MWIR; source DLR FireBird Mission) images from Sept. 24^th^ and Oct. 15^th^, 2015. Location of image extent within study area indicated in the inset. MODIS hotspot data for date concurrent to TET-1 imagery indicated in each image (red dots; source FIRMS collection MCD14).

Efficient and effective fire management is difficult on many levels [6], and a key component for improvement will depend upon the best fire occurrence monitoring system possible. Early detection of small fires, before they have the chance to become fire fronts many kilometers long, will greatly improve firefighting response efficiency. Fig 7 shows a TET-1 image series as fires first detected on Sept. 24^th^, 2015, thereafter spread and connect with one another over the following 2 weeks to become fire fronts over 10 km long. Relying solely upon the MODIS hotspot data, one would have missed detection of the small fires in September. The hotspot dataset then detects less than half of all fires in the October image. The image displays the F06, F07 and F09 fires, which were estimated to have burned an area ranging from 15,547 ha (using TET-1) to 35,417 ha (using Landsat). The most common fire form observed over the entire study area were fire lines (13 out of 16 fires examined), all of which either started as a single point source or from multiple sources. This emphasizes how early detection of small fires before they have the chance to grow into large fire fronts will be very important for improving fire management efficiency as well as effectiveness.

## Conclusions

This study demonstrates the improved fire detection capabilities of TET-1 compared to commonly accepted fire monitoring systems, and how this sensor allows better measurement of temporal and spatial fire dynamics than heretofore possible. TET-1 fire detection capabilities, given the sensor’s higher saturation temperature and finer spatial resolution, are clearly enhanced compared to those of the MODIS system. The MODIS hotspot data have and will continue to provide valuable information on global fire occurrence, playing a very important role in global monitoring of fire activity and analyses of decadal changes in fire occurrence. The FireBird mission offers an opportunity to build upon this system with a more sensitive fire monitoring system capable of providing more detailed, locally-based information on fire occurrence and previously not possible fire dynamic measurements. Early fire detection through smoke and haze provides valuable information for fire control management. The costs incurred, both financial as well as social, by the fall 2015 fire catastrophe present clear motives for improving current fire control management systems.

Another goal of this study was to provide a first estimate of the damage incurred to the Sebangau National Park during this event. Our calculations of active fire and area burned within the study area range between 225,469.44 ha (TET-1) and 684,561.47 ha (Landsat). This discrepancy is due to differences in detection methodology, where TET-1 is providing snapshots of active fires while Landsat is providing a combined estimate of where fires burned. Both systems used in synergy with one another would support a monitoring system capable of accurately estimating area burned as well as measuring fire dynamics closer to real-time than previously possible. Monitoring of fire damage extent using sensors working in the VNIR and SWIR, such as those used in the Landsat and recently joining Sentinel-2 missions, provide estimates of burned area at fine spatial resolution (down to 10 m) but are limited by the requirement of waiting for relatively cloud and haze free images [34,64]. The longer the period of time between a fire event and acquisition of a clear image, the more vegetation regrowth and resettlement inhibits accurate detection of burn scars [64]. Fire dynamic measurements revealed maximum propagation speeds in excess of 500 m/day and that fires tended to spread most quickly over peat > 2m deep. Based on peat fire propagation speeds measured in other regions, we conclude that this group was likely a mix of quick-moving surface peat fire fronts and slow-burning, low intensity sub-surface peat fires. Changes in vegetation type were observed to co-occur with fire spreading boundaries. We also found that fires occurred with the highest frequency in concession areas containing drainage infrastructure but were not yet cleared prior to the fire event. Fires were observed to often begin in areas previously burned and then spread into neighboring primary forest. While these observations were statistically inconclusive, this demonstrates how the TET-1 sensor offers a wealth of data for further fire dynamic investigations. Conservation efforts, such as the installation of dams, likely helped to minimize spread of fire in some areas, but enhanced fire monitoring systems would provide an integral tool for improving firefighting management.

TET-1 has been joined in 2016 by BIROS, expanding the FireBird constellation and thus reducing time between acquisition dates. Issues with determining burned area from active fire detection data, where fast-moving fire fronts produce a discontinuous time series of events, will be lessened through decreasing the time period between detection events. Future expansion of the FireBird fleet is in discussion, which would enable near real-time fire detection. This would support firefighting activity organization through focusing efforts on fires while they are still small and more easily contained. In this study we have demonstrated not only how a FireBird sensor can improve hereto existing monitoring systems, but also how detected fire dynamic data can be used to help design measures to reduce risk of fire. This information will be useful for government agencies, fire managers and monitoring groups concerned with preventing such catastrophes in the future.
